# Statistical Researches Relative to the Etiology of Pulmonary and Rheumatic Diseases, &c.

**Published:** 1841-10

**Authors:** 


### REVIEWS.

---

Art. IV.—*Statistical Researches relative to the Etiology of Pulmonary and Rheumatic Diseases, illustrating the application of the Laws of Climate to the Science of Medicine; based on the records of the Medical Department of the Adjutant General's office.* By Samuel Forry, M. D., Medical Staff of the U. S. Army, &c. 1840, pp. 44.

The confederated colonies and the United States together, have had an army and a medical staff, for more than 60 years; and the officers of that staff have been stationed in almost every part of our widely extended country, from Passamaquoddy Bay to the Balize, from Jamestown to the Chippewa mountains, from Mackinas to Cape Florida. Thus dispersed, and generally surrounded by new objects, no other portion of the American profession have had opportunities equal to theirs for making new and interesting observations, on the influence of climatic and topographical peculiarities, on the constitutions, of at least one class of society—adult males. It must be confessed, however, that the aggregate of what they have contributed, is by no means worthy of them. For a long time pleasure and dissipation absorbed the time and attention of too many of these gentlemen, not less than their brethren of the line. We are happy to know, that for the last 12 or 15 years this dissipation, like that of the profession in civil life, has been steadily going out of fashion; and that a spirit of professional observation, and habits of scientific inquiry, have, *pari passu*, sprung up. Much of this is, we believe, attributable, not merely to an abatement of army conviviality, but to the establishment at Washington, of the office of a Surgeon General; and the judicious arrangements of Dr. Joseph Lovell, who so long held that important station. Among these, was the order to keep meteorological registers at the different military posts. They were, however, by no means, supplied with all the requisite instruments; and hence but little exact information could be acquired, except on the temperature of those places. Under the direction of the present surgeon General, Dr. Thomas Lawson, these observations, for a period of 10 years, have been collected, arranged and published, under the title of Army Meteorological Register, no copy of which has, however, reached us. This labor was performed by Dr. Forry, and he tells us that after having performed it, he determined on preparing the little work we are now reviewing. It is based upon the tables of that Register, in connexion with the returns, in the Adjutant General's office, of the sick at the corresponding posts, through the long period just mentioned. As the title of the pamphlet before us indicates, our author has limited himself to pulmonary and rheumatic diseases, being those which, beyond all others, seem to depend most immediately on climatic influences. It is our design to make such an analysis of this work, as may be practically useful to our readers.

We have said that all the *exact* meteorological information presented, relates to temperature; nevertheless our author has united with it the element of moisture, although not in possession of any hygrometric tables.

If this continent were destitute of lakes, it might be safely affirmed, that in going from the sea board into the interior its climate would constantly become drier; but with a chain of deep and extended lakes stretching half across it, the humidity of the interior, in their neighborhood, is greatly increased, while at the same time they exert a decisive influence on the extremes of atmospheric temperature along their margins. Under these views our author has established several general divisions of the United States; and recognized different systems of climate in each, founded on the relative distance from the sea and the lakes. His first general division, the northern, extends from Canada as far south as New York, in which, referring to moisture as well as heat, there are three varieties of climate, that of the New England coast, that near the lakes, and that remote both from them and the sea. The second, or middle division extends from New York to Savannah and westwardly to the Rocky Mountains; which division, being without lakes, has but two varieties of climate —the maritime atlantic, and the interior. The third or southern division, embraces, first, the delta of the Mississippi, with the gulf shore, to Pensacola; and second, the peninsula of East Florida, the whole of which is humid, and, in its temperature, uuder the influence of the sea.

The extreme range of the thermometer throughout all the systems of climate in the northern general division, are greater than in those further south, and the difference between the *mean* heat of the winter and summer is greater. But the mean summer and winter temperatures, in the same latitudes in this division, vary according to the nearness or remoteness of different places, to the sea and the lakes. Thus, places near the Atlantic and lake coasts, have not only greater humidity, but warmer winters and cooler summers; for the obvious reason, that masses of deep water do not become so hot in summer, nor cold in winter, at the surface, as tracts of continents, because from their agitation they are warmed to a greater depth. Therefore, the extremes of temperature are greater in the interior, between the lakes and the maritime coast and beyond the lakes, than elsewhere in this division.

The modifying influence of the sea coast compared with the region beyond the lakes, is exhibited in the following table. It presents an average of five years, calculated from the data of two posts in each system of climate, the mean latitude of the posts on the ocean being 43°18', and that of those in the opposite locality, 43°10'.

| Locality. | Latitude. | Mean Annual Temperature. | Range of the Thermometer | | | Winter. | | | Spring. | | | Summer. | | | Autumn. | | |
|---|---|---|---|---|---|---|---|---|---|---|---|---|---|---|---|---|---|
| | | | | | | December. | January. | February. | March. | April. | May. | June. | July. | August. | Septemb'r. | October. | November. |
| | | | | | | 33.20 | 24.18 | 26.45 | 34.21 | 44-76 | 55.37 | 63.26 | 68.96 | 67.43 | 59.85 | 50.42 | 39.73 |
| | | | | | | 25.07 | 18.82 | 21.78 | 34.20 | 48.05 | 64.49 | 75.04 | 76.81 | 73.92 | 60.85 | 52.92 | 37.43 |
| Sea-coast | 43°18' | 47°.19 | 98 | -24 | 122 | 27.94 | | | 44.78 | | | 66.55 | | | 50.00 | | |
| Region beyond the Lakes | 43°10' | 48°.99 | 104 | -30 | 134 | 21.89 | | | 48.91 | | | 75.26 | | | 50.40 | | |

In the former, the mean temperature of winter is 6°.05 higher than the latter; that of spring is 4°.13 lower; that of summer is 8°.71 lower; and that of autumn 0°.40 lower. This contrast is more strikingly shown by comparing the difference in the mean temperature of winter and summer, it being on the sea coast 38°.61, and in the opposite locality 53°.37. It is thus apparent, that a classification of climates, having, for its basis mere latitude, is wholly inadmissible; for, although there may be little difference in the mean annual temperature, yet the distribution of heat among the seasons may be extraordinarily unequal.

These facts are still more strikingly illustrated in the following table, which exhibits a comparison between posts on the lakes and those of the same region situated beyond their influence.

| Locality. | Latitude. | Mean Annual Temperature. | Range of the Thermometer. | | | Winter. | | | Spring. | | | Summer. | | | Autumn. | | |
|---|---|---|---|---|---|---|---|---|---|---|---|---|---|---|---|---|---|
| | | | | | | December. | January. | February. | March. | April. | May. | June. | July. | August. | Septemb'r. | October. | November. |
| | | | | | | 23.04 | 16.98 | 19.85 | 27.20 | 39.44 | 52.56 | 58.24 | 67.13 | 63.51 | 55.94 | 47.19 | 36.33 |
| | | | | | | 18.07 | 13.74 | 20-35 | 31.90 | 44.81 | 62.42 | 71.53 | 76.49 | 72.07 | 58.47 | 50.81 | 36.31 |
| Lakes | 46°27' | 42°.22 | 93 | -26 | 119 | 19.96 | | | 39.73 | | | 62.96 | | | 46.49 | | |
| Remote from the Lakes | 44°53' | 46°.47 | 96 | -26 | 122 | 17.42 | | | 46.38 | | | 73.26 | | | 48.53 | | |

It thus appears that the winter of the former, notwithstanding it is 1°.46 north of the latter, has a mean temperature 2°.54 higher, whilst that of summer is 10°.40 lower. In the latter, the mean temperature of spring is 6°.65 higher, and that of autumn is 2°.04 higher. The difference of the mean temperature of summer and winter, making due allowance for difference of latitude, is even greater than in the comparison with the Atlantic coast, that of the Lakes being 43°, and that of the opposite locality 55°.84. In the former region, the prevailing weather is cloudy, and in the latter fair; thus, during the year the proportion of days is—

| | *Fair.* | *Cloudy.* | *Rain.* | *Snow.* |
|---|---|---|---|---|
| Remote from lakes, | 216 | 73 | 46 | 29 |
| Lakes, | 119 | 132 | 67 | 47 |

In the next or middle general division, there are, we have already said, but two varieties of climate, the humid of the sea shore, and the more arid of the interior. The whole of the middle zone, that in which we live, is characterized by variability, while its mean heat is greater, and the extremes between summer and winter less than in the preceding.

The third or southern division, is still less variable, its annual heat is greater, the mean difference between summer and winter is less, and the moisture of the climate like the maritime portions of the two preceding divisions is great.

Let us travel back, and traverse these zones from south to north, on two different paths, one extending from Key West, in East Florida, lat. 24° 33', through Savannah to Maine, lat. 43° 18' along the Atlantic coast,—the other, from the same Key, through Cincinnati, Ohio, to Fort Snelling on the upper Mississippi, in lat. 44° 53'. In ascending the former, every degree of latitude sinks the mean annual temperature about a degree and a half, but increases the difference between the mean heat of summer and winter about one degree and four tenths. In ascending the latter, each degree of latitude reduces the annual temperature, rather more, because we acquire an elevation above the level of the sea, and increases the difference between the winter and summer means, more than two degrees. On both these paths, the occasional variations of temperature become more frequent, sudden and extreme as we advance north, and this, it is *probable*, at a greater ratio in the interior than on the sea-board. Hence it results that the region of the upper Mississippi, possesses a climate of lower mean temperature, and of greater extremes, than that of any other part of the United States, while it is drier than the sea-board or lake-shore, and not perhaps more moist than any other quarter.

From these data we perceive, that an individual who might sojourn on the sea-coast successively from Maine to Florida, would be acted on by a uniform quantity of moisture, but a constantly increasing degree of heat, and a regular decreasing extent and intensity of variation from heat to cold and cold to heat. And that another individual, who might sojourn progressively from Fort Snelling to Key West, would be advancing into a more humid climate, one in which the mean annual heat was increasing at a greater ratio; and the extremes between summer and winter, and perhaps the violence of the occasional variations, diminishing at a greater ratio still than on the sea-board.

Now the object of our author is to ascertain from the sick reports of different military posts, the influence of these modifications of two of the elements of climate, on the respiratory and fibrous organs, in other words, how far they cause or modify pulmonary and rheumatic diseases. He begins with *catarrh*, and presents the results of ten years observations, at forty-five military posts. As the number of troops was very different at different posts, and varied from time to time at each, he has very properly assumed one thousand as the mean strength, and presented the number of cases in comparison with that strength. We extract his table:

*Ratio of Catarrhal Diseases.*

| Divisions. | Systems of Climate. | Latitude. | Diff. between the mean temp. of winter and summer. | Ratio treated per 1000 mean strength. | | | | |
|---|---|---|---|---|---|---|---|---|
| | | | | First Quarter. | Second Quarter. | Third Quarter. | Fourth Quarter. | Annual result. |
| North'n. | 1st Class. Posts on the coast of N. England, | 43° 18' | 38°.61 | 63 | 49 | 36 | 85 | 233 |
| | 2d Class. Posts on N. chain of Lakes, . . . | 46° 27' | 43°.00 | 90 | 62 | 50 | 96 | 300 |
| | 3d Class. Posts N. of l. 39°, and remote from the ocean and inland seas, . . . . | 44° 53' | 55°.84 | 175 | 120 | 86 | 169 | 552 |
| Mid. | 1st Class. From Delaware Bay to Savannah, . | 37° 02' | 32°.99 | 102 | 45 | 23 | 97 | 271 |
| | 2d Class. South-western Stations, . . . . . | 35° 47' | 36°.83 | 122 | 61 | 33 | 78 | 290 |
| Sou. | 1st Class. Posts on lower Mississippi,* . . . | 30° 10' | 24°.39 | 92 | 34 | 26 | 60 | 218 |
| | 2d Class. Posts in Peninsula of Florida, | 24° 33' | 11°.34† | 45 | 24 | 40 | 33 | 143 |
| | Total, | | | 689 | 395 | 294 | 618 | |

This table, which exhibits the annual and quarterly ratios of each system of climate, and serves to elucidate their relations and sequences, affords a beautiful illustration of the etiology of catarrhal affections as connected with the meteorological laws established. Take, for example, the northern division consisting of the first three classes:—On the New England coast, as the ocean modifies the atmospheric temperature the annual ratio treated per 1000 of mean strength, is as low as 233; on the great lakes, where a similar modifying influence is in operation, it is 300; whilst the third class, characterized by the extreme range of the thermometer, has a ratio as high as 552.

It appears from this table that moisture does not favor the production of catarrh—that the first and third quarters of the calendar year, generate nearly an equal number of cases, the first, however, the most—that either of them produces nearly as many as the intervening two quarters—that the second, comprising April, May, and June, originates fewer, than the succeeding three months, and that the proportion of cases diminishes as we advance from North to South, into climates where the annual temperature is greater, and the extremes between summer and winter less.

*For want of a better arrangement, Augusta Arsenal, Georgia, and Fort Mitchell, Alabama, have been included in this class.

†This result is obtained from the observations made at Key West. At Fort Brooke, Tampa Bay, it is 16°.02.

From the similarity, or, we should rather say, identity, of catarrh and bronchitis, the results here given are strictly applicable to the latter; and to what, in common parlance, is called broncial consumption. From these affections our author proceeds, to those of the serous membrane and parenchyma of the lungs. The following is his tabular view:

*Ratio of Pleuritis and Pneumonia.*

| Divisions. | Systems of Climates. | | Ratio treated per 1000 of mean strength. | | | | |
|---|---|---|---|---|---|---|---|
| | | | First Quarter. | Second Quarter. | Third Quarter. | Fourth Quarter. | Annual Results. |
| North'n. | 1st Class. | Posts on the coast of New England, - - | 12 | 11 | 8 | 10 | 41 |
| North'n. | 2d Class. | Posts on Northern chain of Lakes, - - | 11 | 15 | 13 | 11 | 49 |
| North'n. | 3d Class. | Posts on north of lat. 39°, and remote from the ocean and inland seas, - - - - | 14 | 11 | 7 | 12 | 45 |
| Mid. | 1st Class. | Coast from Delaware Bay to Savannah, - | 21 | 11 | 8 | 16 | 57 |
| Mid. | 2d Class. | South-western Stations, - - - - | 46 | 18 | 10 | 20 | 92 |
| Sou. | 1st Class. | Posts on the lower Mississippi, - - - | 20 | 9 | 4 | 11 | 47 |
| Sou. | 2d Class. | Posts in the Peninsula of East Florida, - - | 14 | 9 | 8 | 6 | 39 |
| | | Total, | 138 | 84 | 58 | 86 | |

It appears from this table, *first*, that the number of cases of catarrh and bronchitis is more than five times as great (5.42 to 1.00) as the number of cases of pleurisy and pneumonia: *second*, that the fourth quarter of the year, which produces nearly as many cases of the former diseases as the first, scarcely produces more of the latter, than the second and third; but that the first originates an extraordinary number. In the table on catarrh, the proportion of cases in the first to the fourth, is 689 to 618. In the table on pleurisy and pneumonia the numbers are 138 to 86. Taking the two together, the proportion of cases of these different affections generated in the first quarter, compared with the fourth, is as 827 to 704—from which it is clearly manifest that the change from cold to warm weather, or the vicissitudes of spring, are more productive of pulmonary inflammation, than those of autumn, or the transition from hot to cold weather, which we think is contrary to the common opinion; and seems to show that cold generates a phlogistic diathesis. *Third*, these tables show that pleurisy and pneumonia, unlike catarrh and bronchitis, are not more common in the interior than on the seaboard and lake shore. Thus if we add together the cases which occurred in the north general division, at the posts, upon the two former, they make 90, while those which occurred at the posts beyond the lakes made 45—just half the number. *Fourth*, it is singular that on the lake shores, the second and third, or the hot quarters of the year, are more given to pleurisy and pneumonia than the first and fourth, in the proportion of 28 to 22, while catarrh is more frequent in the latter than the former, in the proportion of 186 to 112. *Fifth*, these tables show us, that while long continued cold and great contrast of the seasons, predispose, relatively, more to bronchial affections, long continued heat predisposes, relatively, more to pleurisy and pneumonia. Thus while the proportional number of cases of the former compared with the latter, on the posts of the upper Mississippi, is as 552 to 45—the proportional number, at the posts on the Arkansas and Red River, is but as 290 to 92; and when we compare New England with East Florida, a similar result is obtained, for in the former, catarrh and bronchitis stand to pleurisy and pneumonia, as 233 to 41; but in the latter, only as 143 to 39; and while the posts on the lower Mississippi and gulph coast, are less subject to catarrh, than the shores of the lakes, they are almost as liable to pleurisy and pneumonia. Why long continued heat should abridge the liability to mucous inflammation, at a greater ratio than that to serous and parenchymatous inflammation, we do not pretend to know. We may doubt, however, whether these returns possess very great exactness. The word pneumonia, often expresses nothing more definite than the phrase inflammation of the lungs. An inflammation of the bronchial membrane, generally spreads into the adjoining cellular tissue, and *vice versa*. In many cases the signs of both affections are present; in others the diagnostic symptoms of neither are made out; the inquiries of the physician going no farther, than to determine the existence of pulmonary inflammation; which may be bronchitis or pneumonia, or both combined.

Dismissing these affections we come to phthisis; and here again we may doubt whether the cases reported under that head, are all tuburcular, as chronic bronchitis, pleurisy, and pneumonia, so often simulate that specific malady. We extract the tabular view:

*Ratio of Phthisis Pulmonalis.*

| Divisions. | | Systems of Climate. | Ratio treated per 1000 of mean strength. | | | | |
|---|---|---|---|---|---|---|---|
| | | | First Quarter. | Second Quarter. | Third Quarter. | Fourth Quarter. | Annual Results. |
| North'n. | 1st Class. | Posts on the coast of New England, - - | 2 | 3 | 2 | 3 | 9* |
| | 2d Class. | Posts on Northern chain of Lakes, - - - | 3 | 2 | 2 | 2 | 9 |
| | 3d Class. | Posts north of lat. 39°, and remote from the ocean and inland seas, - - - - - - | 2 | 1 | 1 | 1 | 5 |
| Mid. | 1st Class. | Coast from Delaware Bay to Savannah, - - | 4 | 5 | 2 | 3 | 13 |
| | 2d Class. | South-western stations, - - - - - | 3 | 3 | 4 | 2 | 11 |
| Sou. | 1st Class. | Posts on the lower Mississippi, - - - - - | 3 | 3 | 2 | 2 | 9 |
| | 2d Class. | Posts in the Peninsula of East Florida. - - | 2 | 2 | 2 | 2 | 9 |
| | | Total, | 19 | 19 | 15 | 15 | |

The *first* remark we make on this table is, that the influence of the seasons is less perceptible, than in the other pulmonary affections, and that the former half of the calendar year occasions a greater number than the latter. The *next* is, that the region beyond the lakes, where the cold is greatest, the contrast of the seasons most violent, and catarrh most prevalent, phthisis is rarest. The *third*, that this disease prevails, relatively, most in the middle grand division, between the latitudes of 31° and 39°. The *fourth*, that on the whole it appears to be more frequent near the water than remote from it. The *fifth*, that it is as frequent in the south as the north—on the peninsula of East Florida, as on the coast of New England, or the Lakes. Results so different from our preconceived opinions might astonish us, if we did not remember, that these observations were made on troops which have been largely recruited in the north, and transported to

*As fractions are not given, and as the mean strength of each quarter varies, the annual results do not always correspond with the total of the quaterly ratios.

the south, taking with them a predisposition to the disease; but while we recognize this fact in favor of southern climates, we cannot shut our eyes to another, which is, that going to the south when predisposed to consumption does not, of course, avert the development of the malady. Our author is even of opinion, that the febrile diseases of the south tend to develope tubercle. This is doubtless the case in those, who go from the north, predisposed to that heterologous secretion; but facts are wanting to show that malaria, or the acute diseases arising from it, can generate a tubercular diathesis.

The work before us presents a tabular view of the relative mortality, from different pulmonary affections, in the great climatic divisions, which we shall pass over, as their mode of termination is so much modified by the treatment, which, not being given, is not before us. On the whole we may, however, admit with our author, that these diseases, when they do occur, are more complicated and fatal in the south than in the north; and that many persons laboring under chronic pulmonary affections, have their lives shortened, by attempting a *permanent* residence in a hot climate. Their constitutions are enfeebled by the long continued action of a hot atmosphere, laden with miasmata, to neither of which had they been accustomed. But, in which of the different forms of pulmonary disease, is a sojourn in the south most beneficial? If we may judge by the foregoing table, in the bronchial—in catarrhal consumption or chronic bronchitis. For, compared with pleurisy, pneumonia and phthisis, catarrh is less frequent in the south than in the north. He then, and he only, who has a bronchial or laryngeal consumption, should expect to be cured by going to the south; and even he should not remain there in the summer, but seek a cooler climate, and a purer air. Aged persons who are liable to winter coughs, with dyspnœa, stricture and copious expectoration, would be particularly benefitted by escaping from the north in October, and not returning till May.

But where within the United States should they go? May they stop in South Carolina, Georgia, Alabama, Mississippi or

Louisiana? We think not. The vicissitudes in winter are too great; the depression of temperature for short periods often too low; the number of cold rains too many; the domestic means of protection against a damp and chill atmosphere too few and incompetent, to render a winter sojourn within the limits of those states, efficient, as a means of cure. We admit that in reference to climate only, *something* may be gained, by those laboring under bronchial affections, but in numerous cases it is fully counteracted, by sinister influences, from which the patient would be exempt if he had remained at home.

It is only on the peninsula of East Florida, far south, in the latitude of 26° or 24°, that a climate is found of sufficient elevation and constancy of temperature, to exert a really curative influence. Whatever may be the humidity of the air in that latitude, it is always mild. There is, indeed, but little difference between summer and winter. Sudden and violent changes of temperature are unknown, and the atmosphere is always soft and balmy. But in reference to the fitness of this climate for invalids we must let our author speak for himself.

In treating of the climate of Florida, the primary object held in view is, to direct attention to its fitness as a *winter* residence for northern invalids. In 1833, Professor Dunglison called the attention of the profession, on the strength of the meteorological registers kept by the medical staff of the army, to the suitableness of St. Augustine and Tampa Bay as a winter retreat. An examination of abstract No. 2, of Appendix, showing—1. The mean temperature of each month, each season, and the whole year; 2. The difference between the mean temperature of each month and season; and 3. The annual and monthly ranges of temperature—will, it is believed, not only furnish further confirmation of the doctrines already conclusively established, but lead to results of great value to the practical physician.

The results of the four posts in abstract No. 2 of Appendix, illustrate the modifying agency of large bodies of water. Fort King, situated in the interior, has a warmer summer and a colder winter than the remaining stations, all of which are on the coast. Athough Key West is 4°39′ south of Fort King, and has a mean temperature of 3°.43 higher, yet the mean summer temperature is 2°.81 lower. The equalizing influence of the ocean is still further shown by the annual range of the thermometer, the mean of the monthly ranges, and the mean difference of successive seasons. During the summer months, the morning and evening observations are nearly the same at both points, the disparity being caused by the exalted temperature of Fort King at mid-day. In each year, July was the hottest month at Key West, and the month of June at Fort King. As is usual in southern latitudes, there is little variation presented at Key West in the mean temperature of the same month in different years.

There is little difference between the thermometrical phenomena presented at Key West and the Havanna. Within the period of six years, (from 1830 to 1835 inclusive,) the mercury at Key West was never known to rise higher than 90°, nor sink lower than 44°.

The peculiar character of the climate of Florida, as distinguished from that of more northern latitudes, consists less in the mean annual temperature than in the manner of its distribution throughout the year. At Fort Snelling, Iowa, the mean temperature of winter is 17°.29, and of summer, 72°.80, whilst at Fort Brooke, Tampa Bay, the former is 65°.02, and the latter 81°.04 and at Key West, 70°.05 and 81°.39. Notwithstanding the winter at Fort Snelling is 52.76 colder than at Key West, the summer, at the latter is only 8°.59 warmer. In like manner, although the mean annual temperature of Petite Coquille, La., is nearly 2° lower —that of Augusta Arsenal, Ga., nearly 8°—and that of Fort Gibson, Arkansas, upwards of 10° lower than that of Fort Brooke; yet at all, the mean summer temperature is higher. Between Fort Snelling on the one hand, and Fort Brooke and Key West on the other, the relative distribution of temperature stands thus:—Difference of the mean temperature of summer and winter at the former 55°.51, and at the two latter 16°.02 and 11°.34; difference of the mean temperature of the warmest and coldest month, 61°.18 compared with 17°.68 and 14°.66; and the mean difference of successive months stands as 10°.20 to 2°.97 and 2°44. At Fort Snelling, the annual range of the thermometer is 116°, and at Fort Brooke and Key West it is 56° and 37°; and the mean of the monthly ranges bears the relation of 50° to 29° and 16°. The attention is thus directed merely to a few prominent points, as a reference to the tabular abstract will enable any one to trace much farther this remarkable equality in the distribution of temperature among the seasons in Florida. A comparison with the most favoured situations on the continent of Europe, and the islands held in the highest estimation for mildness and equability of climate, affords results no way disparaging. A comparison of the mean temperature of winter and summer, that of the warmest and coldest months and that of successive months and seasons, furnishes results generally in favour of the peninsula of Florida. The mean difference of successive months stands thus:—Pisa 5°.75, Naples 5°.08, Nice 4°.74, Rome 4°.39, Fort King 4°.28, Fort Marion, at St. Augustine, 3°.55, Penzance, England, 3°.05, Fort Brooke, 2°.97, Key West 2.44 Madeira 2°.41. The mean annual range thus:—Fort King 78°, Naples 64°, Rome 62°, Nice 60°, Montpellier, 59°, St. Augustine 59°, Fort Brooke 56°, Penzance 49°, Key West 37°, and Madeira 23°.* The island of Madeira is esteemed by Dr. Clark as best adapted to consumptive patients.

In the West India islands the mean annual temperature near the sea is only about 80. At Barbadoes, the mean temperature of the season is—winter 76°, spring 79°, summer 81°, and autumn 80°. The temperature is remarkably uniform; for the mean annual range of the thermometer, even in the most variable of the islands, is only 13°, and in some it is not more than 4°.† Contrast this with Fort Snelling, Iowa, which gives a range of 116!

It has been seen that the meteorological agents which determine the ratio of pulmonic lesions, causing the first and fourth quarters to present the highest averages, and the third the lowest, is the marked distinction of season characterized by extremes of temperature. Hence the apparent exception to this rule in the system of climate pertaining to East Florida, where the third quarter has a higher ratio than the second or fourth, (see Table p. 21,) instead of contradicting a general law, corroborates it. As Florida is an ever-green land, the influence of the seasons does not impress the pulmonary organs sufficiently to derange their functions by their transition. Hence the ratio of pulmonic lesions is low; and as the causes which are secondary in excessive climates, grow here into primary ones, these diseases may be as rife in the summer as in the spring or autumn.

*Contrary to the numerical ratios furnished in his tables, Dr. Clark says that "the mean annual range of temperature is only 14°"—an error which has crept into the writings of Dr. Dunglison.

†According to the British army statistics.

The state of the weather as indicated by the course of the winds and the proportion of fair and cloudy days, calculated for the same years as abstract No. 2 of Appendix, is shown in the following table:—

| Places of Observation. | Course of Winds. | | | | | | | | Prevailing. | Weather. | | | Prevailing. |
|---|---|---|---|---|---|---|---|---|---|---|---|---|---|
| | N. | N.W. | N. E. | E. | S. E. | S. | S. W. | W. | | Fair. | Cloudy. | Rain. | |
| | Days. | Days. | Days. | Days. | Days. | Days. | Days. | Days. | | Days. | Days. | Days. | |
| St. Augustine, | 1.55 | 2.86 | 9.08 | 1.03 | 10.83 | 1.11 | 2.64 | 1.33 | S. E. | 19.02 | 5.19 | 6.22 | Fair. |
| Fort King, | 1.62 | 2.79 | 3.46 | 3.54 | 4.37 | 5.63 | 5.96 | 3.08 | S.W. | 25.75 | 2.88 | 1.89 | Fair. |
| Fort Brooke, | 1.53 | 3.72 | 5.58 | 2.89 | 4.44 | 2.75 | 6.42 | 3.17 | S.W. | 20.33 | 4.47 | 5.64 | Fair. |
| Key West, | 3.20 | 3.13 | 10.50 | 5.37 | 5.37 | 0.54 | 1.67 | 0.38 | N. E. | 21.54 | 3.08 | 5.92 | Fair. |

The want of hygrometrical observations to indicate the actual or comparative humidity of the atmosphere is to be regretted. That the air is much more humid than in our more northern regions is sufficiently cognizable to the senses. The dews, even in the winter, are generally very heavy. To guard against the oxidation of metals, as for example surgical instruments, is a matter of extreme difficulty. During the summer, books become covered with mould, and keys rust in one's pocket. *Fungi* flourish luxuriantly. The writer has known a substance of this kind to spring up in one night, and so incorporate itself with the tissue of a woolen garment as to render separation impracticable. As general relaxation and lassitude are consequent on this prevailing humidity, it may exercise some agency in the production of the comparatively high ratio of pulmonic and rheumatic affections in the summer season. One of the best safeguards against its effects is, to wear flannel next the skin—a custom generally adopted in the army. It is, indeed, a hygienic measure no less valuable in warm than in cold climates, affording comparative immunity against thermometrical and hygrometrical vicissitudes.

As the rains, however, generally fall at a particular season, so the atmosphere in winter is comparatively dry and serene. The following abstract of the monthly fall of rain at Key West, is the mean result of five years observation:—

| *Jan.* | *Feb.* | *March.* | *April.* | *May.* | *June.* | *July.* | *Aug.* | *Sep.* | *Oct.* | *Nov.* | *Dec.* | *Annual average.* |
|---|---|---|---|---|---|---|---|---|---|---|---|---|
| 1.82 | 1.34 | 1.98 | 1.09 | 6.34 | 2.39 | 2.84 | 3.30 | 4.35 | 3.33 | 1.49 | 1.13 | 31.40 |

It will be observed that during six months, from November to May, which is the longest period that a northern invalid should remain in this climate, the proportion of rain is but 8-84 inches. It has been already remarked that in tropical climates a portion of the year is known as the rainy season, and that the same quantity descends in a much shorter space of time than in the temperate zone; and that consequently the proportion of fair days and clear skies is infinitely in favour of the former. In the table of the weather just given, in which the ratios are monthly averages, this result is strikingly manifested. At Fort King, the annual number of fair days is 309, whilst on the northern lakes, it is only 119. On the coast of Florida, however, the average is not more than 250 days.

The influence of temperature on the living body is often indicated more accurately by our sensations than the thermometer. The advantages of climate as regards its fitness for the pulmonic, not unfrequently depend on the mere circumstance of exposure to, or shelter from cold winds. The frequency and severity of the winds at St. Augustine constitute a considerable drawback on the benefits of the climate. The chilly northeast blast, surcharged with fogs and saline vapours, sweeping around every angle of its ancient and dilapidated walls, often forbids the valetudinarian venturing from his domicil. To obviate these disadvantages, a large house was erected at Picolata on the St. John's; but during the pending Indian disturbances, it has been converted into a barrack and an hospital.

To persons labouring under an irritable state of the bronchial membrane, high winds are particularly injurious. If the consumptive invalid have much sensibility to harsh and keen winds, and if the immediate vicinity of the sea be known to disagree, Fort King ought to be recommended before St. Augustine or even Fort Brooke; but as sea-air is known to be generally adapted to a relaxed habit and a languid and oppressed circulation, a favorable position on the coast should, in such cases, be selected as a winter residence.

The natural advantages of position, without reference to extrinsic circumstances are now under discussion. St. Augustine is on the eastern coast; Fort Brooke is at the head of Tampa Bay,* about 30 miles from the Gulf of Mexico; Fort King is intermediate to these two points; and Key West belongs to the Archipelago south of Cape Sable. The pending

*The old Spanish appellation was Espiritu Santo, or bay of the Holy Ghost, the name Tampa being then restricted to an arm.

hostilities with the Aboriginies, and the difficulty of obtaining accommodations indispensable to the comfort of the invalid, render a winter retreat almost impracticable any where but at St. Augustine or Key West. The oldest town in the United States, and built mostly of a concrete shelly stone in the Spanish style, St. Augustine presents an antiquated appearance, enlivened by beautiful orange groves bending at due season beneath their golden fruit.* The old Spanish fort and the *tout ensemble* of the city, give it more the resemblance of a place of defence than of elegance; but it is these circumstances which associate it with history, and render it peculiarly interesting to the American traveller. Moreover, the inhabitants extend kindness and hospitality towards strangers. Key West, about 60 miles southwest of Cape Sable, is two miles wide and ten long, and is remarkable as being the most southern settlement in the United States. Possessing a good harbor, it has been from time to time the station of our West India squadron. It contains about 1400 souls, and is a place of commerce chiefly in the way of wrecked goods.

In contemplating the scenery of East Florida in the month of January, one is apt to forget that it is a winter landscape. Fort Brooke on Tampa Bay is a truly delightful spot, in which tropical fruits flourish luxuriantly. The lime, the orange, and the fig find there a genial soil, whilst the moss-covered† live-oak and the Pride of China, add beauty and variety to the scene. Vegetation is continuous through every season, culinary vegetables growing in January; and the temperature of the waters in rivers and bays will admit of bathing in every month of winter. To the northern man, all nature seems changed. Even the birds of the air—the pelican and flamingo—indicate to him a climate entirely new. Having accompanied a boat expedition, the object of which was to explore the sources of the St. Johns, the writer found in the month of January, the high cane-grass which covers its banks intertwined with every variety of morning glory, (*convolvulus.*) The thermometer at mid-day, in the shade, stood at 84° Fahr. and in the sun, rose to 100°; and at night we pitched no tents, but lay beneath the canopy of heaven with a screen perhaps

---

*In 1835, nearly all the orange trees in Florida were destroyed by frost—an occurrence previously unknown in the memory of the oldest inhabitant.

†This parasite, (*Tilandsia usneoides,*) known by the vulgar name of Spanish moss, casts a sombre aspect over the scenery of Florida. Resembling the weeping willow clothed in the garniture of hoary age, it has been styled, not inappropriately, the shades of death.

over the face as a protection against the heavy dews. Notwithstanding the day attains such a high temperature, the mercury just before day-light often sinks to 45°, causing a very uncomfortable sensation of cold.

But there are other localities which combine advantages equally important to the pulmonary invalid. On the eastern coast of Florida, at New Smyrna for example, the warmth and softness of the air wafted from the isles of the West Indies across the gulf-stream, in the winter months, are truly grateful to the senses lulling them into repose. Even the virtuoso would not be without materials for contemplation; for here may be seen the ruins of Dr. Turnbull's colony of Greeks, Italians, and Minorcans—his unfinished castle, whose dilapidated and mouldering walls are covered with ivy, amid the luxuriance of the palm,* orange, mangrove, and magnolia. Cape Sable and the coast extending northward towards Key Biscayno, as well as the adjacent islands, would also afford an excellent winter retreat. Adapted to the cultivation of tropicoid fruits, and abounding in game, fish and turtle, this region, from the prevalence of the sea-breeze or trade-winds, presents a climate delightful even in the summer season. Key Biscayno, which is situated on the southeastern coast of Florida, affords an excellent harbor of safety and protection from the storms which frequently rage along the coast. The island is thus described by a medical officer of the army, when stationed there six months:—

"In the midst of summer, the constant prevalence of the sea-breeze renders it at all times delightful in the shade. During the winter, frost is never known; nor is it ever so cold as to require the use of fire. The eastern beach commands a beautiful view of the open sea, and offers, especially during low tide, an admirable place for exercise on horseback for the distance of four or five miles, and for morning and evening walks. The waters around abound in green turtle, and a variety of excellent fish, forming a wholesome and nutritious diet, particularly well suited to cases of pulmonary disease. There is also an abundance of crawfish and crabs. The mainland is only a short distance off, abounding with deer and a variety of other kinds of game, affording a fine field for the

---

*The cabbage-palm, [*chœmarops palmetto,*] is a beautiful tree, presenting sometimes a straight column of 80 feet without a limb. The trunk is generally enclosed by the foot-stalks of the old branches, resembling a coarse net-work. The embryo head is esculent, bearing the taste of unripe chesnuts. The leaf is used in the manufacture of hats, mats and baskets, as well as in the construction of the Indian's wigwam.

sport and exercise of hunting; and the vicinity of the West India islands will, at all times, present the opportunity of procuring the best of tropical fruits.

"The proprietor of the island will, in a short time, erect buildings, and will establish every means in his power for the convenience and comfort of those who may be disposed to visit the place for the recovery of their health. There has been not a single case of fever among the troops since I have been stationed here, and I have no hesitation in stating my opinion that it will be perfectly healthy at all seasons of the year."

When not exposed to the influence of malaria, the climate of Florida, as along the eastern coast, is, even in the season of summer, quite salubrious. The sea-breezes, aided by the deposition of moisture from the atmosphere, generally render the nights pleasant, even in the hottest months and in the centre of the Peninsula. The writer can state from personal knowledge, that after the middle of August the nights become so cool that a blanket is desirable. The climate of the tropics is characterized, as Humboldt justly remarks, much more by the duration of heat than its intensity; and it is to the action of this unceasing high temperature that much of the injurious influence of tropical climes on northern constitutions, is to be ascribed.

But woe to the invalid that braves the torments of a summer residence under the disadvantages of a camp life? Insects are the pest of a tropical clime. As to fleas, flies, and ticks, the interior of Florida may well rival Egypt in the days of Pharoah. The chigoes insinuate themselves beneath the skin, where they soon establish populous colonies. Flies seem, indeed, to form a component part of your food, your drink, and the atmosphere you inhale. Lizards, snakes, and scorpions, get into your bed, whilst the industrious ant and weevil not only eat your rations, but devour your books—the food of the mind. All nature seems alive; and every hour you observe some uncouth living thing, whose family name has scarce been registered by the entomologist. In addition to these annoyances, you will have a nightly serenade performed by wolves and alligators—a woful concert of whining yells and dismal bellowings, constituting the realization of a *howling* wilderness.

Since a more rational view of the nature and causes of pulmonary diseases has prevailed, the beneficial effects of change of climate in certain forms have been fully established. Formerly, when consumptive patients were indiscriminately condemned to endergo expatriation, the unfortunate invalid often sank before he reached his destination, or he was doomed soon to add another name to the long and melancholy list of his countrymen, who seem to have sought a foreign land, far from friends and home, only to find a premature grave. When it is considered, however, that all remedial agents have proved so inefficacious in phthisis pulmonalis as to place it emphatically among the *opprobria medicorum*, it is no ways surprising that its victims should seek beneath the influence of a more genial clime, the relief, however uncertain, denied them in their own.

The south-western coast of a country is generally mild and humid, and consequently soothing but rather relaxing. In diseases accompanied with an inflammatory condition of the general system, or dependent on an excited state of particular organs, this variety of climate has been found more especially beneficial. Decided advantage may reasonably be anticipated in chronic inflammatory affections of the trachea and bronchia, attended with a day cough and little expectoration; but when such cases occur in individuals of a languid and relaxed state of constitution, accompanied by copious expectoration from the mucous surfaces, the disease is much more likely to be aggravated than relieved. These remarks, which are made by Clark "on Climate," are equally applicable to all other diseases attended with great relaxation of the general system. It is, therefore, manifest that, in recommending a change of residence to invalids, attention to these distinctions, both in regard to varieties of climate and peculiarities of disease, is absolutely necessary.

The climate of Florida has been found beneficial in incipient cases of pulmonary consumption, and those threatened with the disease from hereditary or acquired predisposition. It is in chronic bronchial affections more particularly that it speedily manifests its salutary tendency. To distinguish the *bronchial* from the *tubercular* form of the disease, often demands considerable powers of discrimination; and upon this distinction frequently hangs the propriety of a removal to a southern clime. The application of the physical means of exploration, now so ardently cultivated, has fortunately given a greater degree of certainty to our diagnosis.

But there are other forms of disease in which such a climate as that of East Florida is not unfrequently of decided advantage. To this class belongs *asthma*. As this term is too commonly applied to every disease in which difficulty of respiration is a prominent symptom, let us not prescribe for a mere name; but when consulted on the propriety of a change of climate, let the pathological condition of the patient be duly estimated. In simple spasmodic asthma, unconnected with organic disease, or in that form which is complicated with chronic bronchitis, or is symptomatic of primary irritation in other viscera, such as the stomach, intestines or uterus, the patient is generally much benefited. In asthma connected with affections of the heart, a mild climate often affords temporary relief. In this variety of complication, a sea-voyage is frequently of striking service.

In chronic disorders of the *digestive* organs, when no inflammation exists or structural changes have supervened in viscera important to life, but the indication is merely to remove diseases of a functional character, a winter residence promises great benefit; but exercise in the open air, aided by a proper regimen, are indispensable adjuvants.

In many of those obscure affections, called *nervous*, unconnected with inflammation, exercise and travelling, in this climate, are frequently powerful and efficient remedies.

*Chronic rheumatism*, though apparently much less under the influence of meteorological causes than pulmonic affections, will often be benefitted by a winter residence in Florida. As these cases often resist the best directed efforts of medicine, it is the only remedy which the northern physician can recommend with a reasonable prospect of success. When the disease is complicated with much derangement of the digestive organs, it is customary in Europe to visit such places as combine the additional advantage of a course of bathing, as the mineral waters of the Pyrenees, those of Aix in Savoy, and the various baths of Italy.

When there exists a general delicacy of the constitution in childhood, often the sequel of rubeola or scarlatina, manifesting itself by symptoms indicative of a scrofulous disposition, a winter residence in a warm climate frequently produces the most salutary effects. At the period of puberty in females, a similar condition of the system often arises, preventing the development of those new functions peculiar to this stage of life. This general derangement, if not soon corrected, often results in a constitutional disorder beyond the resources of our art, denominated by Clark "*Tubercular Cachexy*," the precursor of pulmonary consumption. If the winter can be passed in a warm climate, and the patient have the advantage of exercise on horseback, warm sea-bathing, and a well regulated diet, the youthful invalid may often be rescued from an untimely grave.

Another form of disease remains to be alluded to, in which a change of climate promises its healing powers, *viz:* premature decay of the constitution, characterized by general evidence of deteriorated health, whilst some tissue or organ important to life commonly manifests symptoms of abnormal action. This remarkable change often occurs without any obvious cause, and is not inappropriately termed in common parlance, "a breaking up of the constitution."

Let not the invalid, however, trust too much to a change of climate. Unfortunately for the character of the remedy, it has been recommended indiscriminately and without proper consideration. It has been too often resorted to as a last resource or forlorn hope; or in cases susceptible of alleviation or permanent cure, it has been wholly misapplied. One person is hurried from his native land with the certainty of having his sufferings increased and his life shortened, instead of being allowed to die in peace in his own family; whilst another, who might derive much advantage from the change, is sent abroad wholly uninstructed in regard to the selection of a proper residence, or ignorant of the various circumstances by which alone the most suitable climate can be rendered beneficial. It is one of our most powerful remedial agents, and one too which, in many cases, will admit no substitute. But much permanent advantage will result neither from travelling nor change of climate, nor their combined influence, unless the invalid adheres strictly to such regimen as his case may require. This remedy must be considered in the light of all other therapeutic means, and to ensure its proper action, it is necessary that the requisite conditions be observed.

At the present time, St. Augustine and Key West are the only places, which afford the conveniences required by the wants of an invalid; but assuming that proper accommodations can be equally obtained at all points, Key Biscayne on the southeastern coast, or Tampa Bay on the Gulf of Mexico, claims a decided preference, especially over St. Augustine. As a general rule, it would be judicious for the northern physician to direct his pulmonary patient to embark about the middle of October for Tampa Bay. Braving the perils of the wide ocean, he will realize the healthful excitement incident to the fears and hopes of a sea-voyage. Having spent the winter months at Tampa, let him proceed early in March to St. Augustine, by way of Dade's battle-ground and the old Seminole agency. In addition to the corporal exercise, he will find food for mental digestion at every step of his journey. Having thus reaped the benefit of a sea-voyage and all the advantages to be derived from a change of climate, the valetudinarian may return to his anxious friends so much renovated in health and spirits as to be capable of enjoying again the blessings of social life.

As long, however, as predatory Seminole bands retain possession of this peninsula, few itinerant invalids will imitate the example of the celebrated Spanish adventurer, Ponce de Leon, who, in the wild spirit of the 16th century, braved the perils of unknown seas and the dangers of Florida's wilds, in search of the far-famed fountain of rejuvenescence. When the period, however, of the red man's departure shall have passed, the climate of this "land of flowers" will, it may be safely predicted, acquire a celebrity as a winter residence not inferior to that of Italy, Madeira, or Southern France.

A paragraph of this extract it will be observed, relates to the influence of the climate of Florida on *rheumatics*. It is not improbable, that in many coses chronic rheumatism may be alleviated or cured by a sojourn in the south; but we must confess that the evidence in favour of this opinion, furnished by the work before us is not very strong. From a table which we shall not extract, the proportion of cases which have occurred at the posts of East Florida, is greater than the posts of New England, and scarcely less than an average of all the military stations in the union. It is, however, considerably less than the average of the garrisons near the lakes and on the upper Mississippi; though, again, it is greater than the posts of the south west and the lower Mississippi, especially the last, where the number is only about two thirds of the general average; from all which we are warranted in cautioning our brethren, against promising their patients with chronic rheumatism, the same benefits from a visit to East Florida, which they may predict for those who labour uuder chronic laryngitis and bronchitis.

In conclusion we must express our gratification at the care, ability and candour with which Dr. Forrey has discharged the duty assigned him; and express the hope that our army friends will persevere in the good work they have begun, until the medical statistics of the army shall assume a scientific and settled character; and the results of their enquiries be made available, in practice, to the whole medical profession. D.

